# Prognostic accuracy of frailty & vulnerability screening in older ED patients: a systematic review & meta-analysis

**DOI:** 10.1093/geroni/igaf122.2378

**Published:** 2025-12-31

**Authors:** Nai-Wen Ku, Yu-Chi Hsu, Jasmine Mudhur, Lusine Abrahamyan, Chu-Lin Tsai, Alibhai Shabbir, Martine Puts

**Affiliations:** University of Toronto, Toronto, Ontario, Canada; National Taiwan University Hospital, Taipei, Taipei, Taiwan (Republic of China); University of Toronto, Toronto, Ontario, Canada; University of Toronto, Toronto, Ontario, Canada; National Taiwan University Hospital, Taipei, Taipei, Taiwan (Republic of China); University Health Network, Toronto, Ontario, Canada; University of Toronto, Toronto, Ontario, Canada

## Abstract

Frailty is associated with increased mortality and morbidity in older adults, highlighting the need for effective screening in emergency departments (EDs). However, the optimal tool for predicting adverse outcomes remains uncertain. Therefore, our review’s question is: which frailty and vulnerability screening instrument has the highest prognostic accuracy for adverse outcomes in older adults visiting EDs? Studies were eligible for inclusion if they included patients aged ≥60 admitted to the ED, used frailty or vulnerability instruments, and reported sensitivity, specificity, and area under the curve (AUC). Databases searched until January 25, 2025, included MEDLINE, EMBASE, Cochrane Library, CINAHL, and CNKI. Study quality was assessed using the updated Quality in Prognosis Studies (QUIPS) tool. Two investigators independently reviewed abstracts and full-texts. A meta-analysis was conducted for tools with consistent cutoffs in ≥ 4 studies. Sixty-seven papers described 62 studies using 40 screening instruments. The Identification of Seniors at Risk (ISAR) was the most frequently used (n = 25), followed by the Clinical Frailty Scale (CFS) (n = 15) and the Triage Risk Screening Tool (TRST)(n = 11). Most instruments showed high sensitivity but low specificity. Meta-analysis was feasible for ISAR and TRST. ISAR’s pooled sensitivity, specificity, and AUC for in-hospital mortality were 92% (95% CI, 88-95), 26% (95% CI, 21-33), and 0.82. For ED revisits, ISAR’s AUC was 0.64, while TRST’s was 0.60. Frailty screening instruments in EDs exhibit high sensitivity but low specificity, limiting clinical utility. Future research should enhance specificity by incorporating domains beyond geriatric conditions and known prognostic indicators into frailty instruments in large-scale studies.

